# RNA interference is not involved in natural antisense mediated regulation of gene expression in mammals

**DOI:** 10.1186/gb-2006-7-5-r38

**Published:** 2006-05-09

**Authors:** Mohammad Ali Faghihi, Claes Wahlestedt

**Affiliations:** 1 Department of Biochemistry, The Scripps Research Institute, 5353 Parkside Drive, Jupiter, FL 33458, USA; 2 Center for Genomics and Bioinformatics, Karolinska Institutet, Berzelius väg, SE-171 77 Stockholm, Sweden

## Abstract

**Background:**

Antisense transcription, yielding both coding and non-coding RNA, is a widespread phenomenon in mammals. The mechanism by which natural antisense transcripts (NAT) may regulate gene expression are largely unknown. The aim of the present study was to explore the mechanism of reciprocal sense-antisense (S-AS) regulation by studying the effects of a coding and non-coding NAT on corresponding gene expression, and to investigate the possible involvement of endogenous RNA interference (RNAi) in S-AS interactions.

**Results:**

We have examined the mechanism of S-AS RNA base pairing, using thymidylate synthase and hypoxia inducible factor-1α as primary examples of endogenous genes with coding and non-coding NAT partners, respectively. Here we provide direct evidence against S-AS RNA duplex formation in the cytoplasm of human cells and subsequent activation of RNAi.

**Conclusion:**

Collectively, our data demonstrate that NAT regulation of gene expression occurs through a pathway independent of Dicer associated RNAi. Moreover, we introduce an experimental strategy with utility for the functional examination of other S-AS pair interactions.

## Abstract 2

The study of two examples of endogenous genes with coding or non-coding natural antisense transcript partners provides evidence against the involvement of RNAi in the natural antisense-mediated regulation of mammalian gene expression.

## Background

Naturally occurring antisense transcripts (NAT) have been reported for 20% of the human genome [1-3]. Recent reports indicate the existence of NAT for at least 72% of mouse transcripts [4,5]. Most NAT are *cis*-encoded antisense RNA [6,7]. By definition, *cis*-NAT are complementary mRNA with an overlapping transcription unit at the same chromosomal locus. *Trans*-NAT are complementary RNA transcribed from different chromosomal locations [8]. Chimeric transcripts are mRNA with identity to more than one region of the genome and might be an artifact of cDNA library production [9]. Over 70% of *cis*-NAT have a tail-to-tail format with a 3' overlap, while 15% have a head-to-head format with a 5' overlapping region. The remaining molecules have intronic or coding sequence overlaps [10]. Many NAT show no open reading frame and are, therefore, classified as non-coding RNA [11-13].

The interaction between antisense and corresponding sense transcript partners does not follow a unified and predictable pattern [4]. Here, we investigated the interactions between two well-validated NAT targeting the human genes encoding hypoxia inducible factor-1α (HIF) and thymidylate synthase (TS). The antisense transcript for HIF (aHIF) is a non-coding RNA that may alter HIF splicing and also the ratio between the two splice forms of HIF [14-16]. Specifically, it has been hypothesized that the antisense molecule may destabilize one splice variant of HIF mRNA and shift the balance in favor of the other variant [17,18]. Editing is another proposed function of NAT through transformation of the adenosine to inosine nucleotide in pre-mRNA. The antisense sequence for TS (rTSα) induces editing of the sense RNA molecule, and thereby drives TS mRNA down-regulation [19,20]. Importantly, the NAT for TS is protein coding, whereas there are no predicted open reading frames for aHIF. Thus, we chose to study these two known candidates from coding and non-coding subgroups of NAT, which could potentially modulate sense mRNA through two distinct modes of action.

One of the most exciting findings in genome biology in recent years has been the discovery of RNA interference (RNAi), which has been proposed as a possible mechanism by which NAT may regulate gene expression [9,21]. RNAi is an innate cellular process activated when a double-stranded RNA (dsRNA) enters the cell. Originally discovered in *Caenorhabditis elegans*, RNAi is an evolutionarily conserved, post-transcriptional gene silencing mechanism. The dsRNA is processed by the RNase III enzyme called Dicer into small duplex RNA molecules of approximately 21 to 22 nucleotides, termed small interfering RNA (siRNA). The siRNA molecules then interact with a multi-protein complex, termed RNA-induced silencing complex (RISC), resulting in sequence specific association of the activated RISC complex with the cognate RNA transcript. This interaction leads to sequence-specific cleavage of the target transcript [22]. It has been suggested that dsRNA derived from endogenous sense-antisense (S-AS) duplexes may act through the RNAi pathway by serving as a substrate for Dicer, and the subsequent generation of siRNA. The siRNA would then regulate one or both of the S-AS transcripts [9,23].

In summary, NAT have been proposed to regulate gene transcription, RNA splicing, polyadenylation, editing, stability, transport, and translation [24]. The aim of this study was to explore the mechanism of NAT action. Shared complementary regions in exons of NAT imply the probability of cytoplasmic duplex formation, and intronic overlap sequences suggest that they form nuclear dsRNA duplexes. In theory, all proposed regulatory mechanisms would require RNA duplex formation in the cytoplasm or nucleus; therefore, cellular evidence for RNA duplexes, using HIF and TS as model genes, were the main focus of this work.

## Results

**Figure 1:**
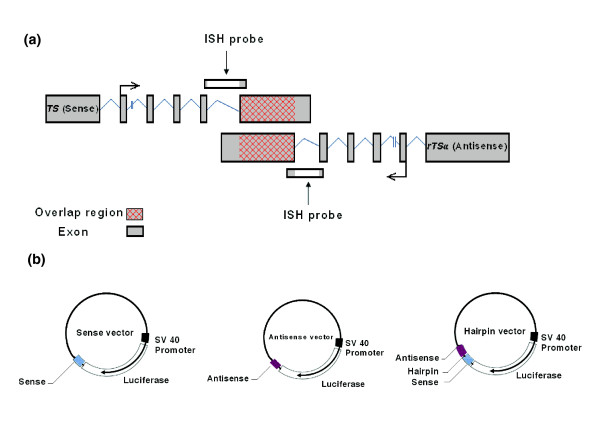
Thymidylate synthase genomic location. **(a) **Schematic presentation of TS sense and rTSα antisense mRNA. Exons are presented as boxes and the location of probes used for *in situ *hybridization (ISH) as well as the 3' overlap region of both sense and antisense mRNA are also indicated. The entire overlap region of both sense and antisense mRNA (red hash shaded region) were cloned into the vector described in (b). **(b) **Conformation of vectors used for transfection and S-AS RNA production. The sense vector makes luciferase RNA with a 3' sense overlap sequence, the antisense vector makes an analogous RNA with a 3' antisense overlap region, and the S-AS vector makes RNA with a consecutive sense-antisense sequence with a hairpin sequence between them.

**Figure 2:**
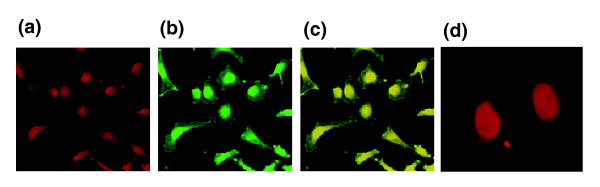
Single cell RNA expression of TS transcripts. **(a) **Antisense probe; **(b) **sense probe; **(c) **both sense and antisense probes bound to the fixed and reverse transcribed TS RNA in HeLa cells. Probes were designed to cover exon boundaries and a part of the overlap region in a strand specific manner. **(d) **Signals from the actin probe show that the method was working optimally. All the probes were intron spanning to avoid background signal from contaminating DNA.

The *in situ *hybridization method was used to assess the simultaneous presence of both endogenous TS and rTSα. HeLa cells were grown on the surface of slides, fixed and treated with DNase (see Materials and methods). First strand cDNA was synthesized and subjected to *in situ *hybridization using strand specific intron spanning probes (the schematics for the TS sense-antisense gene and probes are illustrated in Figure 1a). Importantly, the use of intron spanning probes eliminate detection of contaminating DNA, and the probes covered at least a portion of the overlap region for both transcripts, ensuring that the signals were obtained from a full mRNA. The reverse complementary probe was used for detection of RNA transcripts, before first strand cDNA synthesis, and produced the same pattern of signal distribution with less intensity (data not shown). Our results show both transcripts co-exist in single cells at the same time (Figure 2).

**Figure 3:**
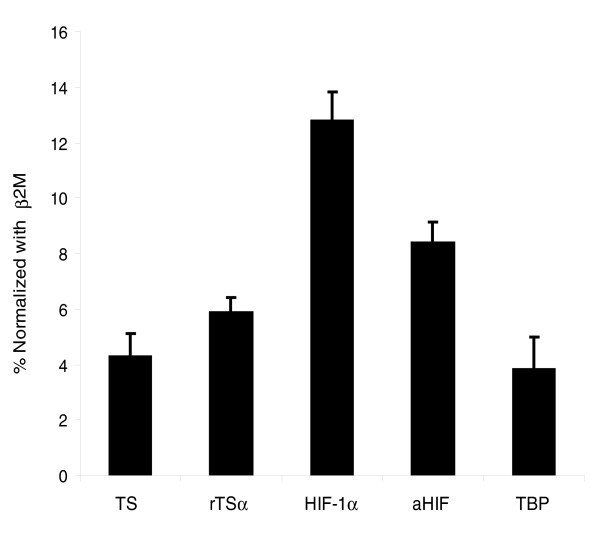
Endogenous single cell expression of TS sense (TS) and its antisense (rTSα) mRNA, as well as HIF sense (HIF) and its antisense (aHIF) RNA. Real-time PCR primers were designed to span between the overlapping and non-overlapping regions. Expression of the low abundant TATA box binding protein (TBP) was also quantified to determine the sensitivity of the assay. All samples were normalized to β2M and the average results from 15 individual cells are plotted.

To demonstrate the co-existence of S-AS pairs in single cells, as opposed to cell populations, we designed a method to detect the co-expression of NAT within a single cell. We extracted RNA from a single cell, under microscopic guide, for the quantification of S-AS transcripts by real-time PCR using TaqMan technology (Figure 3). Primers were strand specific for sense and antisense RNA of both genes. We normalized S-AS expression to a highly abundant mRNA, β2-microglobulin (β2M), as an internal control. We also gauged the sensitivity of our methods by comparing the expression of TS, rTSα, HIF and aHIF with that of a relatively low abundance gene product, TATA binding protein (TBP). The S-AS mRNA expression levels were 5% to 13% of that of β2M, as expected for genes with low expression, and TBP levels were 5% relative to β2M levels (Figure 3). Thus, both S-AS transcripts were present in single cells at approximately similar levels.

**Figure 4:**
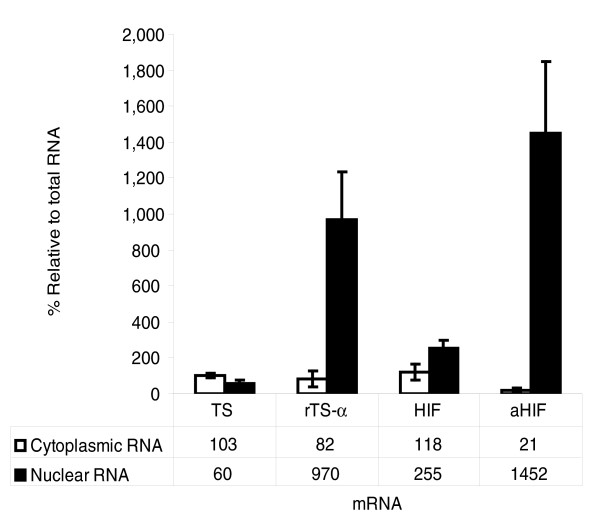
Cellular localization of TS sense (TS) and its antisense (rTSα) RNA and HIF sense (HIF) and its antisense (aHIF) RNA in three cell lines (HeLa, S-KN-MC and HEPG2). The cytoplasmic and nuclear RNA were normalized to total RNA and graphed as the average for three cell lines.

We next investigated the cellular location of TS and HIF transcripts. Cytoplasmic and nuclear extracts were prepared from HeLa cells and immediately used for RNA extraction. RNA was then reverse transcribed and used for quantification of S-AS transcripts by real-time PCR. Importantly, the sense strands of both genes had similar expression levels in the cytoplasm and nucleus; in contrast, antisense transcript levels were 1,000-fold higher in the nucleus compared with the level detected in the cytoplasm. These data thus suggest a spatial dissociation in S-AS pairs (Figure 4).

**Figure 5:**
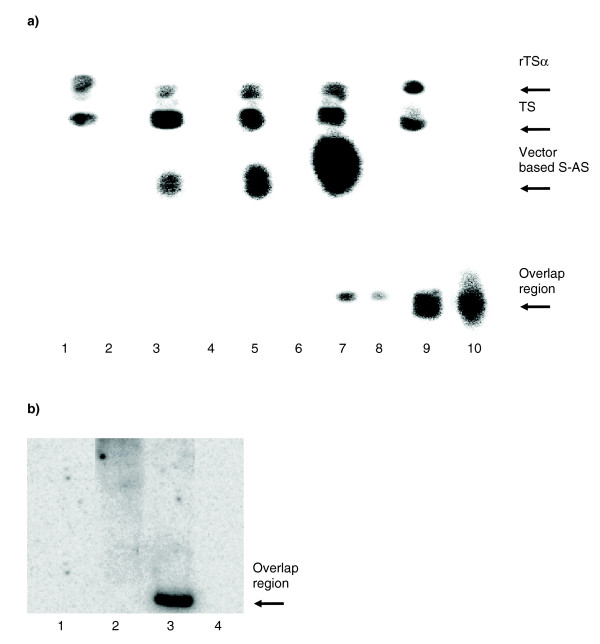
Duplex RNAs were not detected in HeLa cells using RPA. **(a) **Ribonuclease protection assay (RPA). of cytoplasmic RNA. Lane 1, HeLa lysate -RNAse; lane 2, HeLa lysate +RNAse; lane 3, HeLa overexpressing sense (S) -RNAse; lane 4, HeLa overexpressing sense (S) +RNAse; lane 5, HeLa overexpressing antisense (AS) -RNAse; lane 6, HeLa overexpressing antisense (AS) +RNAse; lane 7, HeLa overexpressing hairpin vector (S-AS) -RNAse; lane 8, HeLa overexpressing hairpin vector (SAS) +RNAse; lane 9, HeLa transfected with *in vitro *transcribed S-AS RNA duplex -RNAse; lane 10, HeLa transfected with *in vitro *transcribed S-AS RNA duplex +RNAse. All of the +RNAse samples treated with RNAse A+T, along with -RNase samples, were separated on denaturing PAGE and probed for the overlap region of TS mRNA. The predicted positive bands (rTSα, 1,800 bp endogenous antisense mRNA; TS 1,600 bp endogenous sense mRNA and 1,100 bp vector based S-AS mRNA) were detected in RNAse negative samples and revealed efficacy of RNAse treatment as well as specificity of the probe. Additionally, signals corresponding to a 200 bp product (protected overlap region) were seen only in the last four lanes, which had synthetically endogenous or exogenous RNA duplex. **(b) **Additional controls for RPA of cytoplasmic RNA. Lane 1, cytoplasmic lysate of HeLa cells; lane 2, cytoplasmic lysate of HeLa cells overexpressed with sense and antisense vector; lane 3, lysate from HeLa cells transfected with *in vitro *transcribed S-AS RNA duplex; and lane 4, total RNA from HeLa cells overexpressing sense and antisense vector. All samples were treated with RNAse A+T, separated on denaturing PAGE and probed for overlapping region of TS mRNA. The expected 200 bp product (protected overlapping region) was seen only in lane 3, which included exogenous synthetic RNA duplex.

Next, we explored the formation of S-AS duplexes in the cytoplasm of HeLa cells using the ribonuclease protection assay (RPA). Although HeLa cells endogenously express both sense and antisense mRNA, we constructed three vectors that produce sense, antisense or consecutive S-AS overlapping mRNA in eukaryotic cells (Figure 1b). For two of the constructs, the 3' overlap region of TS and rTSα were placed downstream of a luciferase gene, thereby allowing transfection efficiency to be monitored. For the third construct, we engineered both the sense and antisense complementary regions in the same vector with a short hairpin between the S-AS overlap parts; this was termed a hairpin vector. RNA from this vector will supposedly fold back on itself to form an RNA duplex in cells, mimicking the repeat regions in the genome, and were used as a positive control. For an additional control, we performed *in vitro *transcription (IVT) of the vectors, made artificial RNA duplexes and then transfected them into the cells. To investigate the presence of RNA duplexes in transfected and untreated cells, cytoplasmic lysate was isolated and subsequently treated with RNAse A and T prior to separation on a polyacrylamide gel. Existing RNA duplexes were detected with radiolabeled probes for the S-AS overlap regions. As expected, S-AS duplexes were detected in cells transfected with IVT dsRNA and in cells transfected with the third vector (hairpin vector) designed to make a synthetic hairpin RNA duplex. Additionally, endogenous S-AS single-stranded RNA, as well as vector based RNA, were detected in RNAse negative samples. In cells overexpressed with sense, antisense or cells expressing endogenous levels of NAT, RNA duplexes were not detected (Figure 5a). RNA duplexes were not detected even in the cytoplasm of the cells overexpressed with both sense and antisense vectors at the same time (Figure 5b). These data suggest that endogenous NAT, as well as synthetically overexpressed S-AS RNA, did not form duplexes in the cytoplasm of HeLa cells.

**Figure 6:**
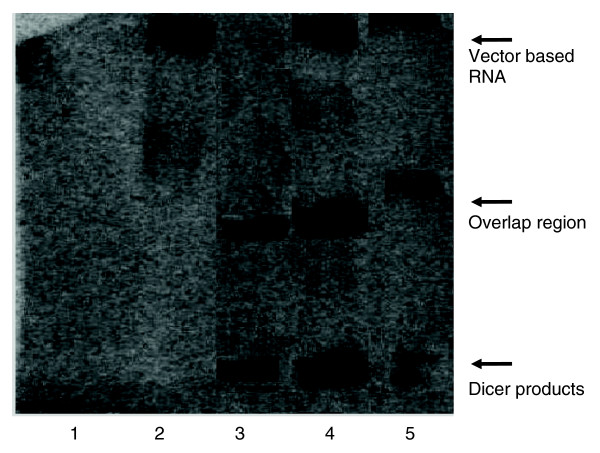
Northern blot for Dicer products. Total RNA from: lane 1, HeLa cells; lane 2, HeLa cells overexpressed with S-AS mRNA; lane 3, HeLa cells transfected with IVT-overlap dsRNA; lane 4, HeLa cells overexpressing hairpin S-AS RNA; lane 5, marker. The vector based RNA (1,100 bp) band in lanes 2 and 4 represent mRNA originating from the vector. The overlap region (200 bp) band in lane 3 is the transfected overlap RNA, and the same band in lane 4 could represent an intermediate product from siRNA production or a byproduct of the cell interferon response. The Dicer product (approximately 20 bp) band represents 21 nucleotide RNA sequences, characteristic of RNAse III enzyme products.

It is possible that putative RNA duplexes in the living cells are transient and labile and are processed to endogenous siRNA or other intermediate products rapidly. To investigate this possibility, we designed a Northern blot analysis with radiolabeled probes spanning the overlap region of the S-AS mRNA. These randomly designed probes, which can potentially detect S-AS sequences of any length from full length RNA to less than 20 base-pair (bp) Dicer products, were used to search for the presence of processed RNA. The hypothesis was that, if RNA duplexes are present, they should ultimately be processed by Dicer into the 21 bp RNA oligonucleotides. HeLa cells were transfected with the same vectors used in the previously described experiment, which produced sense, antisense, or hairpin RNA. The RNA duplexes from the S-AS overlap region produced by IVT served as a positive control and were transfected into the cells. Dicer products were only present in cells transfected with IVT dsRNA or cells transfected with a hairpin vector, which produced internal hairpin dsRNA (Figure 6). As seen in the previous experiment, hairpin vector produces an RNA duplex due to the vicinity of the S-AS sequences and it mimics repeat regions in the genome. This observation suggests that, in our experimental setting, the only form of the RNA that could form a duplex and be processed by the endogenous siRNA production pathway is the hairpin form. Positive bands were detected in overexpressed cells at 1,100 bp (full length RNA originating from the vector), as well as at 200 bp in IVT RNA transfected cells. The 200 bp band in the cells transfected with the hairpin vector might be an intermediate product in siRNA processing or, alternatively it could be a byproduct of the cell interferon response. However, the lack of 21 bp RNA molecules in untransfected or overexpressed cells suggests S-AS duplexes were not processed by Dicer.

**Figure 7:**
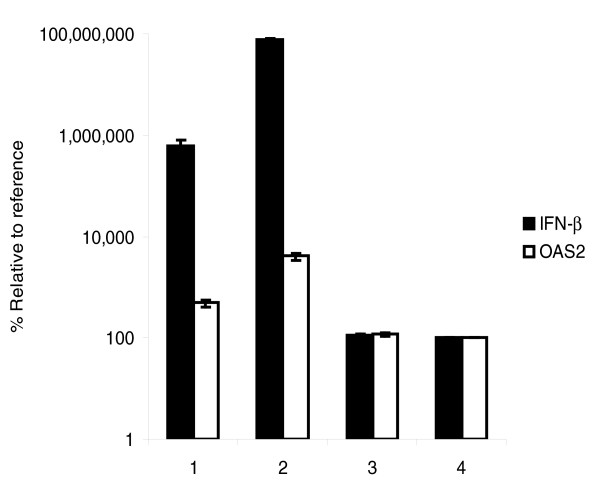
IFNβ and OAS2 mRNA expression. The interferon response was quantified by qRT-PCR in: HeLa cells transfected with IVT-dsRNA of HIF (1) or IVT-dsRNA of TS (2); HeLa cells overexpressing S-AS of TS (3); or reference HeLa cells (4). The mRNA levels were normalized to glyceraldehydes 3-phosphate dehydrogenase. In cells transfected with duplex RNA originated from TS or HIF, there was a remarkable increase in IFN-β and OAS2 mRNA.

The interferon signaling cascade is part of the cell's antiviral defence mechanism and can be triggered by dsRNA. Interferon (IFN)-β and 2',5' -oligoadenylate synthetase-2 (OAS2) mRNA levels were measured in cells overexpressing S-AS transcripts (Figure 7). IFN-β mRNA levels were up-regulated up to 10,000-fold in cells transfected with *in vitro *transcribed dsRNA from HIF or TS but were unchanged in cells with overexpressed S-AS transcripts. OAS2 levels were also up-regulated, by about 500-fold, only in the cells with IVT duplex RNA transfection. These data indicate that cytoplasmic RNA duplexes with S-AS mRNA are unlikely to form; nevertheless, it is possible that the IFN pathway may be unresponsive to intracellular RNA duplexes.

## Discussion

Taken together, the present results suggest that NAT do not form cytoplasmic RNA duplexes that activate RNAi mechanisms. Overlapping transcripts in an antisense orientation, be they protein coding or non-coding, have the potential to form dsRNA, a substrate for a number of different RNA-modification pathways. One prominent route for dsRNA is its breakdown by Dicer enzyme complexes into small RNA. We used several experimental approaches to identify the presence of RNA duplexes in the cytoplasm of cells, and to detect Dicer products involved in processing of dsRNA. Our results, using synthetic S-AS constructs as well as endogenous NAT, did not support the presence of cytoplasmic RNA duplexes or engagement of the RNAi mechanism.

The concomitant presence of both sense and antisense mRNA is one requirement for NAT regulation and many *in silico *predicted NAT candidates can be ruled out on this criterion alone. Expression levels of S-AS pairs are also important, as these could predict the mode of regulation. High levels of S-AS pairs in a single cell, as suggested from our experimental model, argue against RNAi involvement. However, another explanation for this phenomenon is a translation block or other kind of RNA mediated regulation of gene expression, without alteration of mRNA levels. Expression assessment and evaluation of mRNA levels would be recommended as a first step in studying other predicted S-AS candidates.

Alterations in antisense transcript levels can affect the sense mRNA level; however, S-AS changes are not necessarily reciprocal. Recently, we showed that antisense transcript knock down elevated sense transcript levels but the reverse interaction was not observed [4]. This observation suggests antisense mRNA is involved in sense transcript regulation, but sense mRNA does not appear to control antisense expression. If endogenous RNAi were involved in mammalian S-AS phenomena, then it may be expected that both transcripts exhibit similar expression profiles in knockdown experiments.

Overall, the above observations are consistent with the conclusion that RNAi mechanisms are not engaged by S-AS gene regulation. Indeed, further support is derived from two other observations. First, small RNA molecules were not detected even for highly expressed S-AS pairs, implying Dicer-independent RNA processing. Second, the IFN cascade was not activated by NAT. Indeed, it may have been expected that, if at least 70% of mammalian genes have NAT and the mechanism is through RNA duplex formation, there would be a cumulative IFN response. Our studies show a dramatic IFN-β and OAS2 mRNA induction with dsRNA transfection, but not in cells overexpressing S-AS pairs, indicating the absence of duplexes of NAT.

To date, there are no reports of endogenous mammalian siRNA derived from NAT in the literature [25]. It is possible, however, that endogenous siRNA could be programmed into RISC and that this effect would be long term and lead to down-regulation of target RNA. In theory, a 500 bp dsRNA would produce a library of siRNA. This siRNA collection could impair protein production at two levels, either by degrading many 'off targeted' mRNAs or by blocking translation. The extent of this non-specific effect would be much greater when considering the large number of genes known to have antisense sequences. It is worth noting that many research groups have identified and cloned all known small regulatory RNAs, such as miRNA and repeat associated siRNA [26,27]. An interesting observation is that no perfect match RNA oligonucleotides have been reported.

Consistent with data in the present investigation, Jen *et al*. [28] pursued a meta-analysis of NAT expression and suggested that RNA degradation by dsRNA formation is not a predominant route of gene regulation in *Arabidopsis thaliana*. Additionally, endogenous siRNA has been defined for plants; however, only 11 pairs of NAT had siRNA sequences mapped uniquely to the overlapping region of NAT, which substantiates the notion that RNAi is not involved in the processing of S-AS pairs [29]. In other words, although the presence of endogenous miRNA has been reported, no endogenous mammalian siRNA originating from NAT has been described so far. This observation also argues against processing of endogenous RNA duplexes in a Dicer-dependent pathway and further substantiates our findings.

Our data suggest that antisense expression is not linked to transcript degradation pathways. However, our methods do not completely exclude the formation of RNA duplexes in the cell nucleus, or any proposed functions for NAT regulation of gene expression, such as editing, nuclear retention, splicing or transport. Although many different functions and mechanisms have been suggested for NAT, no systematic approaches for the classification or prediction of these mechanisms have been suggested to date. Our study could be a start for a functional approach to NAT studies that could lead to a categorization of NAT based on their unique bioinformatic features. Our methodology could also be expanded to provide a systematic approach to natural antisense mediated regulation of gene expression.

## Materials and methods

### *In situ* hybridisation

HeLa cells were grown on the surface of silane-coated slides overnight and fixed with 4% paraformaldehyde (pH 7.4) for 4 minutes. After air drying of the slides, a chamber was utilized for easy treatment of the attached cells with DNase at 37°C for 16 hours. DNase Master Mix contained 10× TurboDNase Buffer (Ambion Europe, Cambridgeshire, UK), 100 units DNase1, 100 units of TurboDNase, and 100 units of Suprasin in a final volume of 200 μl. The cells were then washed with 1× phosphate-buffered saline (PBS) and subsequently incubated at 95°C for 5 minutes. First strand cDNA was synthesized with an RT-Master Mix of 10× RT buffer (Applied Biosystems, Foster City, CA, USA), 2.5 mM MgCl_2_, 10 mM dNTP mixture, 10 pM random hexamers, 100 units RNase inhibitor, and 500 units of reverse transcriptase in a final volume of 200 μl. The reverse transcription (RT) reactions were completed using the following conditions: 30 minutes at room temperature, 3 hours at 42°C, and 5 minutes at 95°C. For *in situ *hybridization, the cells were incubated at 65°C for one hour in blocking buffer (10 mM Tris-HCl, 50 mM KCl, 1.5 mM MgCl_2_, 1% Triton-X, 20 μM random DNA in a final volume of 200 μl). After blocking, the cells were hybridized at 70°C for one hour with 10 μM of specific intron spanning probes (the sequences are given in Additional data file 1). The slides were then washed two times with pre-warmed PBS. Hybridization of the probe directly to the RNA was done under the same conditions without RT.

### Dilutional single cell real-time PCR

The HeLa cultures were diluted to a few cells in each bright field. RNA was extracted from 15 individual cells that were picked under the guide of a confocal microscope. First strand cDNA synthesis was made from the RNA by using SMART and CDS III 3' oligonucleotides and Powerscript reverse transcriptase from Clontech (Mountain View, CA, USA) according to the manufacturer's instructions. The first strand cDNA was then used for PCR amplification using the LD primer, DSIII PCR primer, and Advantage2 Polymerase mix from the Clontech cDNA library kit.

### Preparation and fractionation of cell extracts

Cytoplasmic extracts were prepared from HeLa cells transfected with different vectors. Cells were harvested after 24 hour transfections and centrifuged at 1,000 *g *for 5 minutes at 4°C. Cell pellets were washed three times with ice-cold PBS, pH 7.2, and lysed for 10 minutes on ice in three packed cell volumes of lysis buffer (20 mM Tris-HCl, pH 7.4, 200 mM NaCl, 14 mM MgCl_2_, 20 units of suprasin, 100 units of protease inhibitor; 100 μg/ml cyclohexamide, 0.1% (v/v) Triton X-100). Nuclei were isolated by centrifugation at 5,000 *g *for 10 minutes at 4°C. The supernatant contained the cytoplasmic extract and was immediately used for RNA extraction with Trizol (Invitrogen, Carlsbad, CA, USA) Nuclear extracts were prepared by washing the pellet once in lysis buffer and twice in 1× PBS, pH 7.2. Nuclear RNA was then collected using Trizol reagent. Purity (>98%) and integrity of nuclei were determined microscopically.

### Ribonuclease protection assay (RPA)

Using the Direct Protect Lysate RPA kit from Ambion, cytoplasmic lysate was treated with RNase cocktail buffer and incubated with RNase A and T cocktail at 37°C for 30 minutes. Nucleases were removed by incubation with sodium sacrosyl and proteinase at 37°C for 30 minutes. RNA was precipitated using 99% ethanol and glycogen blue and subsequently DNase treated with TurboDNase (Ambion) prior to separation on a 5% denaturing PAGE/8 M urea. RNAse negative samples were treated exactly the same, except for addition of RNAse A and T to assess the specificity of the probe and efficacy of RNAse treatment.

### Northern blot for the Dicer products

Total RNA was collected using Trizol (Invitrogen) and precipitated with 99% ethanol. Total RNA (30 μg) was loaded per lane and separated out on a 10% PAGE/urea gel. The RNA was then transferred onto a nylon membrane (Amersham, Little Chalfont, UK) and blocked with salmon sperm DNA for six hours. The blocked membrane was hybridized overnight with radiolabeled S-AS probes spanning the overlap region of the TS and rTSα genes. The probe was made by random priming of overlap DNA using ^32^P-labeled nucleotide and the Amersham random priming kit. All membranes were washed one time with low stringency and two times with high stringency buffer, each for 1 hour, and signal was detected with a Typhoon (Amersham) phosphor-imaging instrument.

### Cell culture and transfection

HeLa cells were cultured in Dulbecco's modified Eagle's medium supplemented with 10% fetal bovine serum. The cells in logarithmic growth were transfected with plasmids containing the luciferase gene with either the sense or antisense overlap region or both. At 24 hours post-transfection, cells were used for further applications. The pGL3 control vector (Promega, Madison, WI, USA) was used for making all S-AS constructs. We engineered Pst1 and EcoR1 restriction sites downstream of the firefly luciferase for cloning. A BamH1 sequence was used to form a hairpin between overlap regions and to construct a vector with a consecutive S-AS sequence (primers and probe sequences are listed in Additional data file 1). The same vector was used as a template for IVT of S-AS overlap mRNA, using a MEGAscript transcription kit (Ambion). For IVT of HIF, transcript primers with a flanking T7 promoter sequence were designed and the PCR product then used for synthesizing duplex RNA.

### Real-time PCR

Real-time PCR was carried out with the GeneAmp 7000 machine (Applied Biosystems). The PCR reactions contained 20 ng cDNA, Sybrgreen or Universal Mastermix (Applied Biosystems), 300 nM of forward and reverse primers, and 200 nM of probe in a final reaction volume of 50 μl (primers and probe sequences are listed in Additional data file 1). The primers and probe were designed using PrimerExpress software (Applied Biosystems). They were strand specific for each S-AS pair and the probe covered exon boundaries to eliminate the chance of genomic DNA amplification. The PCR conditions for all genes were as follows: 50°C for 2 minutes and 95°C for 10 minutes, 40 cycles of 95°C for 15 seconds and 60°C for 1 minute. The results are based on the cycle threshold (Ct) values. Differences between the Ct values for the experimental genes and the reference gene (either β2 M or glyceraldehyde 3-phosphate dehydrogenase) were calculated as ΔΔCt.

## Additional data files

The following additional data are available with the online version of this paper. Additional data file 1 is a table containing sequence information for all the primers and probes used in this study. Primers included were used for real-time PCR, *in situ *hybridization, cloning of Ts and rTSα and for *in vitro *transcription of HIF.

## Supplementary Material

Additional File 1 Primers included were used for real-time PCR, *in situ *hybridization, cloning of Ts and rTSα and for *in vitro *transcription of HIF. Click here for file
