# Supplementary material for: RNA interference is not involved in natural antisense mediated regulation of gene expression in mammals
Source: Genome Biol. 2006 May 9;7(5):R38. doi: 10.1186/gb-2006-7-5-r38 (PMC1779516; doi:10.1186/gb-2006-7-5-r38)
Supplement: Additional File 1 — Primers included were used for real-time PCR, in situ hybridization, cloning of Ts and rTSα and for in vitro transcription of HIF. [file gb-2006-7-5-r38-S1.doc]

**Supplementary Table 1 - Primer and probes used for in situ hybridization, real time PCR and cloning**

| Primer Name | Sequence | Gene |
| --- | --- | --- |
| Actin Probe with 5’ Texas Red | GAAGATCAAGATCATTGCTCCTC | Human β-Actin |
| HIF1A-sense-F | CTGCACAAACTTGGTTAGTTCAATTTT | HIF1α_TaqMan Primer |
| HIF1A-sense -R | ACTGCAATGCAATGGTTTAAATACC | HIF1α_TaqMan Primer |
| HIF1A-sense -P | TTTTTTAGTATGTTCTTTAATGCTGGATCACAGACAGCTC | HIF1α_TaqMan probe |
| antiHIF1A-antisense-F | ATACTCTTTTCAATGGGATATTATGGTTGT | aHIF1α_TaqMan Primer |
| antiHIF1A-antisense-R | TGGTACTGGTTATTTCTACATTTATCTTAGTG | aHIF1α_TaqMan Primer |
| antiHIF1A-antisense-P | TAACATGACATTTAGGGACTCAACATACATTAAGGTGATG | aHIF1α_TaqMan probe |
| HIF-T7IVT-F | TAATACGACTCACTATAGGGTGGTAGCCACAATTGCACA | HIF-*In vitro* transcription |
| HIF-T7IVT-R | TAATACGACTCACTATAGGGCCTGGTCCACAGAAGATGT | HIF-*In vitro* transcription |
| TS-sense Probe with 5’ Fluorescein | GCCACTGAAAATTCAGCTTCA | Thymidylate Synthase |
| TS-Overlap- F | ATCCGCATCCAACTATTAAAATGG | TS-Overlap TaqMan Primer |
| TS-Overlap-R | CCAGCCCAACCCCTAAAGAC | TS-Overlap TaqMan Primer |
| rTS-Antisense Probe with 5’ Texas Red | CCTCAGGAATCAGCTAAAGCAAA | rTSα |
| PstTS antisense-F | aaactgcagAACTTTTACCTCGGCATCCA | TS Cloning primer |
| EcoTS antisense -R | cggaattcAGCGAGAACCCAGACCTTTC | TS Cloning primer |
| EcoTS sense-F | cggaattcAACTTTTACCTCGGCATCCA | rTSα cloning |
| PstTS sense -R | aaactgcagAGCGAGAACCCAGACCTTTC | rTSα cloning |
| E-TS sense-F | cggaattcAACTTTTACCTCGGCATCCA | Consecutive TS S-AS cloning |
| P-TS antisense-F | aaactgcagAACTTTTACCTCGGCATCCA | Consecutive TS S-AS cloning |
| BamH1TS sense -R | cgggatccAGCGAGAACCCAGACCTTTC | Consecutive TS S-AS cloning |
| TS-sense-F | AAAACCAACCCTGACGACAGA | TS_Taqman primer |
| TS-sense-R | GCAGCGCCATCAGAGGAA | TS_Taqman primer |
| TS-sense-P | CATCATGTGCGCTTGGAATCCAAGAGA | TS_Taqman probe |
| rTS-a)antisense-F | GCATTTCAAGTATCCCGTGATG | rTSα _Taqman primer |
| rTS-a)antisense-R | TGTTGAGTAGCCGGGATCCT | rTSα _Taqman primer |
| rTS-a)antisense-P | AGCGGGCTTCCTACATGCCTCCC | rTSα _Taqman probe |
